# Validity and reliability of wireless pressure insoles for measuring gait biomechanics in healthy adults: A protocol for a systematic review and meta-analysis

**DOI:** 10.1371/journal.pone.0336692

**Published:** 2025-11-21

**Authors:** Tomasz Cudejko, Mohammad Al-Amri, Sylwia Szotek, Magdalena Żuk, Magdalena Kobielarz, Kristiaan D’Août

**Affiliations:** 1 Department of Sport, Exercise and Rehabilitation, Northumbria University, Newcastle upon Tune, United Kingdom; 2 College of Biomedical and Life Sciences, Cardiff University, Cardiff, United Kingdom; 3 Biomedical Engineering Department, The Hashemite University, Zarqa, Jordan; 4 Faculty of Mechanical Engineering, Wrocław University of Science and Technology, Wrocław, Poland; 5 Department of Musculoskeletal and Ageing Science, Institute of Life Course and Medical Sciences, University of Liverpool, Liverpool, United Kingdom; The Hong Kong Polytechnic University, HONG KONG

## Abstract

Wireless pressure insoles are emerging as portable, unobtrusive tools for gait analysis in both clinical and real-world settings. These systems incorporate pressure sensors and often inertial measurement units (IMUs), allowing for the collection of spatiotemporal, kinetic, and foot-level kinematic data. Although widely adopted, the measurement properties of wireless pressure insoles—specifically their concurrent validity and test–retest reliability—have not been systematically evaluated. This protocol outlines the methods for a systematic review and meta-analysis that will synthesise the current evidence on the psychometric properties of wireless pressure insoles during walking in healthy adults. This protocol follows the PRISMA-P 2015 guidelines and is registered in the Open Science Framework. We will include peer-reviewed journal articles that assess either the concurrent validity (i.e., simultaneous collection with a gold-standard comparator) or test–retest reliability (e.g., between-day, within-day, or between-rater reproducibility) of wireless pressure insoles during level walking in healthy adults (≥18 years). Outcomes of interest include spatiotemporal, kinematic and kinetic parameters. A comprehensive literature search will be conducted across MEDLINE, CINAHL, Scopus, Web of Science, SPORTDiscus, IEEE Xplore. Screening will be performed independently by two reviewers. Data extraction will follow a pre-piloted template and study quality will be assessed by two independent reviewers using a modified version of the Critical Appraisal of Study Design for Psychometric Articles. Where appropriate, meta-analyses will be conducted using random-effects models, with effect sizes (r, ICC) pooled and heterogeneity assessed via I^2^. This review will provide a comprehensive synthesis of the concurrent validity and test–retest reliability of wireless pressure insoles in healthy adults, offering valuable insights for researchers, clinicians, and technology developers. While methodological heterogeneity may affect the scope of synthesis, the findings will help guide future research and clinical applications.

## Introduction

Gait analysis is widely used to assess human movement in both healthy and clinical populations, offering insights into a broad spectrum of biomechanical outcomes, from simple spatiotemporal measures to more complex kinematic and kinetic variables [1]. Laboratory-based gait analysis systems, such as optical motion capture and force plates, are considered the gold standard for capturing these outcomes. However, these systems are costly, technically complex, and confined to specialized laboratory settings, which limits their accessibility and practicality in everyday clinical practice and real-world applications [2].

Recent advances in wearable technology have led to the emergence of portable and user-friendly alternatives, among which wireless pressure insoles have gained increasing attention. These insoles are embedded with pressure sensors and often incorporate inertial measurement unit (IMU) (e.g., accelerometers, gyroscopes, magnetometers). When worn inside shoes, they can wirelessly collect and transmit biomechanical data during walking without restricting natural movement. Wireless pressure insoles have the potential to measure various gait parameters, including pressure distribution, vertical ground reaction forces (vGRFs), and spatiotemporal variables, both in laboratory [3] and real-world environments [4].

Despite their growing use in research and clinical settings, it remains unclear how reliable and valid wireless pressure insoles are compared to traditional gold standard systems. A number of studies have investigated the concurrent validity of pressure insoles by comparing their outputs to those of 3D motion capture systems, force plates, or instrumented treadmills [5,6]. Similarly, other studies have evaluated their test–retest reliability under repeated measurement conditions [7,8]. However, findings vary depending on sensor type, outcome measured, walking modality, and population. To date, the existing literature reviews on wireless pressure insoles has largely consisted of narrative reviews, with limited emphasis on systematic evidence synthesis and minimal focus on the measurement properties of the technology [9–11].

No systematic review and meta-analysis has yet synthesized the available evidence on the validity and reliability of wireless pressure insoles for measuring biomechanical gait outcomes in healthy adults during level walking. Addressing this gap is crucial to inform researchers, clinicians, and developers about the measurement properties of these systems and to guide best practices in wearable gait analysis. Therefore, we will conduct a systematic review and meta-analysis to determine the i) concurrent validity and ii) test–retest reliability of wireless pressure insoles for measuring biomechanical gait outcomes (e.g., spatiotemporal, kinematic, kinetic) during level walking in healthy adults. The aim of the current protocol is to describe the planned methodology of the review to guide its implementation.

## Methods

### Study design

The study protocol was developed and registered in the Open Science Framework (OSF; osf.io/nfduj/). In accordance with the published protocol, we will conduct a review and document any discrepancies from the protocol. Any modifications will be reflected by updating the OSF record and detailing these changes in a section titled “Differences Between the Protocol and the Review” in the published systematic review results article. Reporting of this protocol adheres to the Preferred Reporting Items for Systematic Review and Meta-Analysis Protocols (PRISMA) guidelines (PRISMA-P checklist- S1 Table) [12]. Reporting of the systematic review will adhere to the PRISMA guidelines for reporting systematic reviews [13]. Systematic review and meta-analysis does not involve collecting data from human beings and hence does not require ethical approval. Patients and public were not directly involved in the design of this protocol, given that the research will examine previously published data. The study is expected to commence in August 2025, with searching, selection and data extraction expected to be completed by December 2025. It is expected that the results from the review will be available in mid-2026.

### Eligibility criteria

Eligibility criteria are based on three domains: types of evidence, study population and outcomes (Table 1).

**Table 1:** Study eligibility.

Domain	Inclusion criteria	Exclusion criteria
Types of evidence	Full-text articles presenting original data with any design theoretically possible, i.e., observational studies (cohort, case-control, cross-sectional) randomize controlled trials randomized trials non-randomized trials	Other types of evidence, i.e.,: literature reviews case studies (n = 1) qualitative studies animal or in-vitro, in-vivo human letters, comments, editorials study protocols, theses, conference papers
Study population	Articles presenting data from healthy adults ≥ 18 years of age	Articles presenting data from participants: with injury, after surgery, with pain, or any health conditions (i.e., musculoskeletal, neurological) that may affect their walking in case of mixed populations, studies that do not stratify results between healthy adults and those having health conditions
Outcomes*	Articles reporting either: the concurrent validity (i.e., simultaneous collection) of biomechanical outcomes during level overground or treadmill walking as measured by the insoles and compared to a gold standard the test-retest reliability (i.e., between-day, within-day, or between-tester; involving the same measure/device/placement with removal between session) of biomechanical outcomes during level overground or treadmill walking as measured by the insoles	Articles reporting: only gait events as outcomes (e.g., timing of initial contact/toe off) • only energy expenditure • only measures of physical activity (i.e., activity counts) • data from real-world, i.e.,home setting, in-clinic, other uncontrolled environment rather than “artificially supervised” setting such as gait laboratory, indoor university etc.

* An included study may pass in reliability criteria, but fail validity criteria (or vice versa).

Wireless pressure insoles will be defined as wearable sensor systems embedded within shoes that wirelessly transmit data and incorporate pressure sensors, and may additionally include IMUs such as accelerometers, gyroscopes, and/or magnetometers. Eligible biomechanical outcomes will include: (i) spatiotemporal parameters (e.g., step time, stride length, stance time), (ii) kinematic parameters (e.g., angular velocity, linear acceleration, orientation), and (iii) kinetic parameters (e.g., vGRFs, peak plantar pressure, trajectory of centre of pressure (CoP)). We will consider the following devices as gold standard comparators: three-dimensional motion capture systems (i.e., stereophotogrammetry), force plates, instrumented mats (e.g., GAITRite), instrumented treadmill. Calibration systems will not be considered independent gold standards, as they do not provide reference data for gait outcomes. Similarly, other wearable systems, including IMUs or alternative insole systems, will not qualify as gold-standard comparators. We will not include studies that aim to train a machine learning model to predict biomechanical outcomes, as these are not designed to evaluate measurement properties of the technology per se.

### Information sources

We will search the following electronic databases: MEDLINE, CINAHL, Scopus, Web of Science, SPORTDiscus, IEEE Xplore. A secondary search will be conducted in: OpenGrey, Google Scholar (first 200 results), and reference lists of all included articles. The search will cover all years up to the final search date. No language restrictions will be applied during the search stage to ensure comprehensive coverage of the relevant literature. The review team is fluent in Arabic, Dutch, English, French, Polish, and Spanish. For articles published in other languages, AI-based translation tools will be used to support screening and data extraction [14].

### Search strategy

The search strategy will be constructed using combinations of three conceptual blocks: (i) measurement system (search terms related to insoles), (ii) outcomes (search terms related to gait biomechanics), and (ii) measurement properties (search terms related to validity and reliability). Search terms will include controlled vocabulary (e.g., MeSH) and free-text keywords and will be tailored to each database. A preliminary search strategy for PubMed is presented in Table 2. Preliminary search strategies for all databases are presented in a S2 Table. Final search strategies will be developed in consultation with a university librarian.

**Table 2:** Draft search strategy in PubMed.

Measurement system	Outcomes	Measurement properties
(“pressure insole*”[tiab] OR “in-shoe pressure”[tiab] OR “instrumented insole*”[tiab] OR “smart insole*”[tiab] OR “smart shoe”[tiab] OR “pressure sensor insole*”[tiab] OR “wearable insole*”[tiab] OR “wireless insole*”[tiab] OR “foot pressure insole*”[tiab] OR “in-shoe system*”[tiab] OR “in-shoe device*”[tiab] OR “in-shoe sensor*”[tiab] OR “pressure-measuring insole*”[tiab] OR “shoe-based sensor*”[tiab]) AND	(“Gait Analysis”[MeSH] OR “Biomechanical Phenomena”[MeSH] OR “Kinetics”[MeSH] OR “gait analysis”[tiab] OR speed*[tiab] OR step*[tiab] OR stride*[tiab] OR cadence*[tiab] OR spatiotemporal[tiab] OR kinematic*[tiab] OR kinetic*[tiab] OR biomechanic*[tiab] OR angle*[tiab] OR acceleration*[tiab] OR force*[tiab] OR load*[tiab] OR “ground reaction”[tiab] OR pressure*[tiab] OR “plantar pressure”[tiab] OR “pressure distribution”[tiab] OR “contact area”[tiab] OR “center of pressure”[tiab] OR “centre of pressure”[tiab] OR gait*[tiab] OR walk*[tiab] OR ambulat*[tiab] OR “stance phase”[tiab] OR “swing phase”[tiab] OR “double support”[tiab] OR joint*[tiab] OR foot*[tiab]) AND	(“psychometrics”[MeSH] OR “reproducibility of results”[MeSH] OR “validation studies as topic”[MeSH] OR “outcome assessment, health care”[MeSH] OR “psychometric”[tiab] OR “psychometric properties”[tiab] OR “measurement properties”[tiab] OR “validity”[tiab] OR “valid”[tiab] OR “reliability”[tiab] OR “reliable”[tiab] OR “reproducibility”[tiab] OR “agreement”[tiab] OR “repeatability”[tiab] OR “test-retest”[tiab] OR “measurement error”[tiab] OR “precision”[tiab] OR “accuracy”[tiab] OR “ICC”[tiab] OR “LoA”[tiab] OR “limits of agreement”[tiab])

### Study records

#### Data management.

All records will be imported into EndNote™ 20 (Clarivate Analytics, Philadelphia, PA, USA) for deduplication and then into Rayyan (Rayyan Systems Inc., Cambridge, MA, USA) for screening. Rayyan is a web-based tool designed to support systematic review screening by enabling blinded, independent screening and conflict resolution among multiple reviewers. Full texts will be stored in a shared folder accessible to all reviewers. Extracted data will be recorded in structured spreadsheets and checked for completeness and accuracy.

#### Selection process.

Two reviewers (TC and KD) will independently screen titles and abstracts. If at least one reviewer deems a title/abstract potentially relevant, the full-text publication will be sought and assessed independently and in duplicate for inclusion. If it is unclear based on the title and abstract whether or not a publication meets the selection criteria, the full-text of the article will be screened. Full-text screening will be independently conducted by two reviewers (TC and MAA), and any disagreements will be resolved by a third reviewer (MK). Prior to title/abstract screening, a calibration activity of 50 publications will be conducted to ensure consistency. Inter-rater reliability for the screening process will be calculated using the kappa coefficient. The screening process, including reasons for excluding full-text records, will be documented using a PRISMA flow diagram.

#### Data collection process.

A standardized data extraction has been developed (S3 Table) and will be piloted on 5 articles prior to extracting data from all articles. One reviewer (TC) will extract all data, and another reviewer (MK) will verify accuracy. Discrepancies will be resolved by discussion. Raw data collected from all eligible studies will be exported to Microsoft Excel (Microsoft Corporation, 365). This data will be made publicly available without any restrictions and will be submitted as part of the Supporting Information Files upon submission of the completed review to a journal.

### Data items

Data will be extracted on: publication details (e.g., author, year, country, funding source, etc.), study details (e.g., aims, study design, population characteristics, sample size etc.), device details (e.g., brand/model of insole, number of sensors, weight, sampling frequency, etc.), details regarding each research question (e.g., specific gait metrics assessed, gold standard comparator, statistical outcomes etc.). Full list of data items that will be extracted is presented in S3 Table. In cases where information is missing or incomplete, the first author (TC) will contact the study authors. Should they be unavailable, available data will be analysed as reported.

### Risk of bias in individual studies

Study quality will be assessed using a modified version of the Critical Appraisal of Study Design for Psychometric Articles [15], which we have adapted to studies evaluating the psychometric properties of the insoles (S4 Table). This tool evaluates 12 items across 5 domains: study question, design, measurements, analyses, and conclusions. Each item is rated from 0–2 and total scores converted to percentage scores. Studies will be rated as: high quality (≥85%), moderate quality (70–84%); low quality (50–69%); very low quality (<50%) Two reviewers (SS & MŻ) will independently assess each study. Disagreements will be resolved by a third reviewer (TC). To reduce bias during the quality assessment phase, raters will be blinded to all identifiable study information. This will be achieved by providing de-identified PDFs of included articles with author names, institutional affiliations, journal names, publication year, and reference lists redacted using PDF editing software.

### Data synthesis

Both narrative (qualitative) and meta-analytic (quantitative) methods will be used to synthesise the results. Quantitative data pooling will follow a multistep classification process: initially, outcomes will be categorised as assessing either validity and/or reliability. These will then be sorted into overarching domains (e.g., spatiotemporal, kinematic, kinetic) and subsequently grouped by specific measures (e.g., step time, stride time, acceleration, peak pressure). As a result, individual studies may contribute to several distinct data groupings based on the type of measurement and the outcomes assessed. Meta-analyses will be performed when at least two studies report the same statistical parameters. Although a range of statistical results will be extracted for qualitative synthesis, quantitative pooling will be limited in advance to Pearson’s correlation coefficients (r) for validity and intraclass correlation coefficients (ICCs) for reliability. Meta-analyses will be conducted using a random-effects model to account for between-study variability. Point estimates will be weighted according to sample size. Due to likely non-normal distribution of r and ICC values, variance-stabilizing Fisher’s z-transformation will be applied prior to pooling. After analysis, pooled effect sizes will be transformed back to their original scales for interpretability. Heterogeneity will be assessed using I^2^ statistic, with thresholds interpreted as: < 25% = low heterogeneity, 25–75% = moderate heterogeneity, 75% = high heterogeneity [16]. Forest plots will be used to visualise pooled estimates, and sensitivity analyses may be conducted to assess the impact of methodological quality or outliers, if sufficient data are available.

Where meta-analysis is not feasible due to heterogeneity in outcomes, metrics, or insufficient data, a narrative synthesis will be undertaken. Additional error metrics — including root-mean-square error (RMSE), standard error of measurement (SEM), minimal detectable change (MDC), and limits of agreement (LoA) — will be extracted and reported descriptively to support interpretation of validity and reliability. The strength of evidence for each outcome will be qualitatively classified as: (i) Strong evidence: consistent findings from multiple high- or moderate-quality studies; (ii) Moderate evidence: consistent findings from multiple studies including at least one high-quality or several moderate-quality studies; (iii) Limited evidence: findings from one high/moderate-quality study or multiple low-quality studies; (iv) Conflicting evidence: inconsistent findings across multiple studies regardless of quality; (v) Very limited evidence: only one low- or very low-quality study available [17].

## Discussion

This systematic review and meta-analysis will critically evaluate the concurrent validity and test–retest reliability of wireless pressure insoles for measuring biomechanical gait outcomes in healthy adults. The review addresses a growing interest in wearable sensor technologies that offer real-world, unobtrusive gait assessment beyond the confines of traditional laboratory environments. Despite their increasing adoption in research contexts, the psychometric properties of wireless pressure insoles have not yet been systematically synthesised and quantified.

A major strength of this review lies in its focused and methodologically rigorous scope. By restricting inclusion to studies comparing wireless pressure insoles with gold-standard gait analysis systems—including three-dimensional motion capture, force plates, instrumented mats, or treadmills—we ensure that only high-quality reference data are used for assessing concurrent validity. This decision allows for a more reliable evaluation of measurement accuracy, avoiding confounding that might arise from comparisons with other wearable systems or calibration tools, which do not independently provide reference gait outcomes. In addition, the review will separately synthesise evidence for concurrent validity and test–retest reliability, offering a comprehensive overview of both how accurate and how stable these devices are over repeated measurements. By doing so, the findings might be relevant for clinicians selecting wearable systems for monitoring patients over time, and for researchers designing longitudinal or intervention studies. The review will also distinguish among spatiotemporal, kinetic, and kinematic parameters, which allows for interpretation across different gait outcomes and device capabilities. Another key strength is the application of a transparent, registered, and reproducible protocol, aligned with PRISMA-P guidelines. The use of a modified psychometric quality appraisal tool, independent duplicate screening, and a pre-specified statistical synthesis plan enhances the objectivity and methodological robustness of the review process. Furthermore, by predefining criteria for evidence strength and including both qualitative and quantitative synthesis (where feasible), the review aims to produce findings that are both rigorous and clinically meaningful.

However, this protocol and its underlying design are not without limitations. First, by focusing solely on healthy adults, we will exclude potentially informative data from studies involving clinical populations. Although this restriction ensures a more homogeneous sample for synthesis, it may limit the generalisability of findings to broader populations. Second, due to the variability in device types, data processing methods, and gold-standard comparators across studies, methodological heterogeneity may preclude some outcomes from being pooled quantitatively. Additionally, some included studies may have used proprietary algorithms or unpublished validation methods, which may affect transparency and comparability. Finally, the exclusion of studies that use other wearable technologies (e.g., IMUs or alternative pressure insoles) or calibration systems as comparators, may also be seen as a limitation. While such devices are commonly used in research, they do not provide independent reference data for spatiotemporal, kinematic, or kinetic outcomes and therefore do not meet our predefined criteria for gold-standard validation. This decision ensures a consistent level of methodological rigor but may limit the number of eligible studies.

Despite these limitations, the review will contribute valuable evidence to guide researchers, clinicians, and developers in understanding the strengths and limitations of pressure insole systems for gait analysis. It will also highlight current gaps in the literature, such as underreported outcomes, under-represented sensor types, or methodological inconsistencies, thereby informing future research priorities. The results of this review will be submitted for publication in a peer-reviewed journal.

## Supporting information

S1 Table PRISMA-P checklist. (DOCX)

S2 Table Preliminary search strategy for each search engine. (DOCX)

S3 Table Data extraction template. (DOCX)

S4 Table Critical appraisal of study design for psychometric articles. (DOCX)
