# Supplementary material for: Validity and reliability of wireless pressure insoles for measuring gait biomechanics in healthy adults: A protocol for a systematic review and meta-analysis
Source: PLoS One. 2025 Nov 21;20(11):e0336692. doi: 10.1371/journal.pone.0336692 (PMC12637928; doi:10.1371/journal.pone.0336692)
Supplement: S2 Table — (DOCX) [file pone.0336692.s002.docx]

| **S2 Table.** Search strategies for each search engine | | | |
| --- | --- | --- | --- |
| Database | Date | N | Search terms |
| MEDLINE  (Pubmed) |  |  | ("pressure insole*"[tiab] OR "in-shoe pressure"[tiab] OR "instrumented insole*"[tiab] OR "smart insole*"[tiab] OR "smart shoe"[tiab] OR "pressure sensor insole*"[tiab] OR "wearable insole*"[tiab] OR "wireless insole*"[tiab] OR "foot pressure insole*"[tiab] OR "in-shoe system*"[tiab] OR "in-shoe device*"[tiab] OR "in-shoe sensor*"[tiab] OR "pressure-measuring insole*"[tiab] OR "shoe-based sensor*"[tiab])  AND  ("Gait Analysis"[MeSH] OR "Biomechanical Phenomena"[MeSH] OR "Kinetics"[MeSH] OR "gait analysis"[tiab] OR speed*[tiab] OR step*[tiab] OR stride*[tiab] OR cadence*[tiab] OR spatiotemporal[tiab] OR kinematic*[tiab] OR kinetic*[tiab] OR biomechanic*[tiab] OR angle*[tiab] OR acceleration*[tiab] OR force*[tiab] OR load*[tiab] OR "ground reaction"[tiab] OR pressure*[tiab] OR "plantar pressure"[tiab] OR "pressure distribution"[tiab] OR "contact area"[tiab] OR "center of pressure"[tiab] OR "centre of pressure"[tiab] OR gait*[tiab] OR walk*[tiab] OR ambulat*[tiab] OR "stance phase"[tiab] OR "swing phase"[tiab] OR "double support"[tiab] OR joint*[tiab] OR foot*[tiab])  AND  ("psychometrics"[MeSH] OR "reproducibility of results"[MeSH] OR "validation studies as topic"[MeSH] OR "outcome assessment, health care"[MeSH] OR "psychometric"[tiab] OR "psychometric properties"[tiab] OR "measurement properties"[tiab] OR "validity"[tiab] OR "valid"[tiab] OR "reliability"[tiab] OR "reliable"[tiab] OR "reproducibility"[tiab] OR "agreement"[tiab] OR "repeatability"[tiab] OR "test-retest"[tiab] OR "measurement error"[tiab] OR "precision"[tiab] OR "accuracy"[tiab] OR "ICC"[tiab] OR "LoA"[tiab] OR "limits of agreement"[tiab]) |
| CINAHL |  |  | ("pressure insole*" OR "in-shoe pressure" OR "instrumented insole*" OR "smart insole*" OR "smart shoe" OR "pressure sensor insole*" OR "wearable insole*" OR "wireless insole*" OR "foot pressure insole*" OR "in-shoe system*" OR "in-shoe device*" OR "in-shoe sensor*" OR "pressure-measuring insole*" OR "shoe-based sensor*")  AND  (MH "Gait" OR MH "Biomechanics" OR MH "Kinetics" OR "gait analysis" OR speed* OR "walking speed" OR step* OR stride* OR cadence* OR spatiotemporal OR kinematic* OR kinetic* OR biomechanic* OR angle* OR acceleration* OR force* OR load* OR "ground reaction" OR pressure* OR "plantar pressure" OR "pressure distribution" OR "contact area" OR "center of pressure" OR "centre of pressure" OR gait* OR walk* OR ambulat* OR "stance phase" OR "swing phase" OR "double support" OR segment* OR joint* OR foot* OR "center of mass" OR "centre of mass" OR "center of gravity" OR "centre of gravity")  AND  (MH "Validity and Reliability" OR MH "Psychometrics" OR MH "Reproducibility of Results" OR "psychometric" OR "psychometric properties" OR "measurement properties" OR "validity" OR "valid" OR "reliability" OR "reliable" OR "reproducibility" OR "agreement" OR "repeatability" OR "test-retest" OR "measurement error" OR "precision" OR "accuracy" OR "ICC" OR "LoA" OR "limits of agreement") |
| SCOPUS |  |  | TITLE-ABS-KEY(("pressure insole*" OR "in-shoe pressure" OR "instrumented insole*" OR "smart insole*" OR "smart shoe" OR "pressure sensor insole*" OR "wearable insole*" OR "wireless insole*" OR "foot pressure insole*" OR "in-shoe system*" OR "in-shoe device*" OR "in-shoe sensor*" OR "pressure-measuring insole*" OR "shoe-based sensor*")  AND  ("gait analysis" OR speed* OR "walking speed" OR step* OR stride* OR cadence* OR spatiotemporal OR kinematic* OR kinetic* OR biomechanic* OR angle* OR acceleration* OR force* OR load* OR "ground reaction" OR pressure* OR "plantar pressure" OR "pressure distribution" OR "contact area" OR "center of pressure" OR "centre of pressure" OR gait* OR walk* OR ambulat* OR "stance phase" OR "swing phase" OR "double support" OR segment* OR joint* OR foot* OR "center of mass" OR "centre of mass" OR "center of gravity" OR "centre of gravity")  AND  ("psychometric" OR "psychometric properties" OR "measurement properties" OR "validity" OR "valid" OR "reliability" OR "reliable" OR "reproducibility" OR "agreement" OR "repeatability" OR "test-retest" OR "measurement error" OR "precision" OR "accuracy" OR "ICC" OR "LoA" OR "limits of agreement")) |
| WEB OF SCIENCE |  |  | TS=(("pressure insole*" OR "in-shoe pressure" OR "instrumented insole*" OR "smart insole*" OR "smart shoe" OR "pressure sensor insole*" OR "wearable insole*" OR "wireless insole*" OR "foot pressure insole*" OR "in-shoe system*" OR "in-shoe device*" OR "in-shoe sensor*" OR "pressure-measuring insole*" OR "shoe-based sensor*")  AND  ("gait analysis" OR speed* OR "walking speed" OR step* OR stride* OR cadence* OR spatiotemporal OR kinematic* OR kinetic* OR biomechanic* OR angle* OR acceleration* OR force* OR load* OR "ground reaction" OR pressure* OR "plantar pressure" OR "pressure distribution" OR "contact area" OR "center of pressure" OR "centre of pressure" OR gait* OR walk* OR ambulat* OR "stance phase" OR "swing phase" OR "double support" OR segment* OR joint* OR foot* OR "center of mass" OR "centre of mass" OR "center of gravity" OR "centre of gravity")  AND  ("psychometric" OR "psychometric properties" OR "measurement properties" OR "validity" OR "valid" OR "reliability" OR "reliable" OR "reproducibility" OR "agreement" OR "repeatability" OR "test-retest" OR "measurement error" OR "precision" OR "accuracy" OR "ICC" OR "LoA" OR "limits of agreement")) |
| SportDISCUS |  |  | TX ("pressure insole*" OR "in-shoe pressure" OR "instrumented insole*" OR "smart insole*" OR "smart shoe" OR "pressure sensor insole*" OR "wearable insole*" OR "wireless insole*" OR "foot pressure insole*" OR "in-shoe system*" OR "in-shoe device*" OR "in-shoe sensor*" OR "pressure-measuring insole*" OR "shoe-based sensor*")  AND  TX ("gait analysis" OR gait OR biomechanic* OR kinematic* OR kinetic* OR speed* OR cadence* OR step* OR stride* OR "walking speed" OR "plantar pressure" OR "pressure distribution" OR "contact area" OR "ground reaction" OR "center of pressure" OR "centre of pressure" OR joint* OR foot* OR segment* OR acceleration* OR force* OR load* OR "center of mass" OR "centre of mass" OR "center of gravity" OR "centre of gravity")  AND  TX (validity OR valid OR reliability OR reliable OR reproducibility OR agreement OR repeatability OR "test-retest" OR accuracy OR precision OR psychometric OR "psychometric properties" OR "measurement properties" OR "limits of agreement" OR ICC OR LoA) |
| IEEE Xplore Digital Library |  |  | (pressure insole OR in-shoe pressure OR instrumented insole OR smart insole OR pressure sensor insole OR wearable insole OR wireless insole OR foot pressure insole)  AND  (speed OR step OR stride OR cadence OR spatiotemporal OR kinematic OR kinetic OR biomechanic OR angle OR acceleration OR pressure OR plantar pressure OR force OR load OR ground reaction OR pressure distribution OR contact area OR center of pressure OR centre of pressure OR gait OR walk OR joint OR foot OR center of mass OR centre of mass OR center of gravity OR centre of gravity)  AND  (validity OR reliability OR reproducibility OR agreement OR consistency OR repeatability OR test-retest OR intra-rater OR inter-rater OR measurement property) |
