# Supplementary material for: Validity and reliability of wireless pressure insoles for measuring gait biomechanics in healthy adults: A protocol for a systematic review and meta-analysis
Source: PLoS One. 2025 Nov 21;20(11):e0336692. doi: 10.1371/journal.pone.0336692 (PMC12637928; doi:10.1371/journal.pone.0336692)
Supplement: S3 Table — (DOCX) [file pone.0336692.s003.docx]

| **S3 Table.** Data extraction template | |
| --- | --- |
| Data items | Associated questions/task |
| *Publication details* | |
| Year | In what year was the study published? |
| First author | Record the first author’s name in the format: Surname Initial (e.g., Gilbert A.) |
| Country | In which country was the study conducted? (Use the first author’s affiliation if the location is unclear) |
| Language | Which language is the study written in |
| Funding source | List any funding agencies or sponsors acknowledged in the study. |
| Role of funder | Was the funder involved in study design, data collection, analysis, or reporting? |
| Data availability | Is the study data publicly available? Yes/No; |
| Data availability | If yes, report the name of the repository. |
| Data availability | If yes, report whether data was raw, semi-processed or fully processed. |
| *General study details* | |
| Study design | What type of study was conducted (e.g., observational, experimental)? |
| Study aim(s) | Validity, reliability or both? |
| Sex | Report percentages of female and male participants. |
| Age | Report mean (SD) for participants’ age |
| Body mass index | Report BMI values (mean, range, SD, if available). |
| Sample size | How many participants were included in the analysis? |
| Sampel size justification | Was sample size justification reported? (yes/o) |
| Measurement setting | Where was data collected (e.g., clinical setting, laboratory, overground track)? |
| Walking velocity | Report the walking speed used during data collection. |
| Walking modality | Was walking performed on a treadmill or overground? |
| Duration of data collection | Report duration of walking (time, n of walking bouts etc) |
| Footwear characteristics | Report footwear type used during data collection |
| *Device characteristics* | |
| Brand and model | Specify the brand and model of the insole system used. |
| Number of sensors | How many sensors were used (e.g., number of pressure sensors, IMU axes)? |
| Sensor type | What type(s) of sensors were used (e.g., capacitive, piezoresistive, piezoelectric)? |
| Device weight | Report the weight of the insole or wearable device, if available. |
| Sampling frequency | What was the sampling rate (Hz) of the system? |
| *Research question 1 - What is the concurrent validity of the insoles during walking in healthy adults?* | |
| Biomechanical outcomes | Spatio-temporal, Kinematics, Kinetics? |
| Statistical outcomes reported | Note all statistical metrics with results that have been reported by authors |
| Gold standard measure | What was used as a gold standard? (report brand and model) |
| *Research question 2 - What is the test-retest reliability of the insoles during walking in healthy adults?* | |
| Biomechanical outcomes | Spatio-temporal, Kinematics, Kinetics? |
| Type of reliability assessed? | Between-day, within-day, or between tester? |
| Time between sessions | Report time between test and retest sessions |
| Statistical outcomes reported | Note all statistical metrics with results that have been reported by authors |
